# AbAgIntPre: A deep learning method for predicting antibody-antigen interactions based on sequence information

**DOI:** 10.3389/fimmu.2022.1053617

**Published:** 2022-12-22

**Authors:** Yan Huang, Ziding Zhang, Yuan Zhou

**Affiliations:** ^1^ State Key Laboratory of Agrobiotechnology, College of Biological Sciences, China Agricultural University, Beijing, China; ^2^ Department of Biomedical Informatics, Key Laboratory of Molecular Cardiovascular Sciences of the Ministry of Education, School of Basic Medical Sciences, Peking University, Beijing, China

**Keywords:** antibody-antigen interaction, deep learning, sequence feature, SARS-CoV, Siamese-like convolutional neural network, webserver

## Abstract

**Introduction:**

Antibody-mediated immunity is an essential part of the immune system in vertebrates. The ability to specifically bind to antigens allows antibodies to be widely used in the therapy of cancers and other critical diseases. A key step in antibody therapeutics is the experimental identification of antibody-antigen interactions, which is generally time-consuming, costly, and laborious. Although some computational methods have been proposed to screen potential antibodies, the dependence on 3D structures still limits the application of these methods.

**Methods:**

Here, we developed a deep learning-assisted prediction method (i.e., AbAgIntPre) for fast identification of antibody-antigen interactions that only relies on amino acid sequences. A Siamese-like convolutional neural network architecture was established with the amino acid composition encoding scheme for both antigens and antibodies.

**Results and Discussion:**

The generic model of AbAgIntPre achieved satisfactory performance with the Area Under Curve (AUC) of 0.82 on a high-quality generic independent test dataset. Besides, this approach also showed competitive performance on the more specific SARS-CoV dataset. We expect that AbAgIntPre can serve as an important complement to traditional experimental methods for antibody screening and effectively reduce the workload of antibody design. The web server of AbAgIntPre is freely available at http://www.zzdlab.com/AbAgIntPre.

## Introduction

Antibody-mediated immunity is an essential part of the immune system in vertebrates. Antibodies are a special class of proteins with Y shape. One key responsibility of these proteins is the specific recognition and neutralization of foreign agents. The root of the specificity of antibodies to a particular antigen can be traced to the diversity of each tip of antibodies’ Y-shaped structures (1). This binding specificity of antibodies has been widely used in the biotechnology and biopharmaceutical industry, where monoclonal antibodies (MAbs) have become the most promising therapeutic method in the market because of their high specificities and long half-lives (2–4). With the rapid advances in bioengineering, more MAb derivatives with greater affinity and specificity are available such as antibody-drug conjugates (ADCs) and fusion proteins (5).

One latest application of MAbs is for the treatment of coronavirus disease 2019 (COVID-19). The COVID-19 pandemic has placed a heavy burden on society. Currently, there are a variety of vaccine strategies, such as inactivated vaccines, nucleic acid-based vaccines, and vector vaccines, to provide protection against COVID-19 (6). All the vaccine strategies aim at enabling the immune system to produce antibodies that bind to the antigens from severe acute respiratory syndrome coronavirus 2 (SARS-CoV-2), the viral pathogen causing COVID-19. But some patients may not be suitable for vaccination due to a severe allergic reaction or may fail to mount a protective immune response through vaccines (7, 8). Therefore, a shortcut turns out to be directly treating the COVID-19 patients with the specific MAb against SARS-CoV-2. Indeed, very recently, anti-SARS-CoV-2 MAbs, including Bebtelovimab and Tixagevimab plus cilgavima, have been approved by FDA for treatment or pre-exposure prophylaxis against COVID-19, suggesting MAbs can become an effective complement to vaccines (9, 10). On the other hand, some other MAbs fail to obtain FDA authorization because of their reduced efficiency against the current Omicron variant of COVID-19 (11), which again stressed the importance of the recognition of antibody-antigen specificity.

Given the importance of identifying the antibody-antigen recognition specificity, radioimmunoassay (RIA) and enzyme-linked immunosorbent assay (ELISA) methods have been widely applied to identify the affinity and specificity of antibody-antigen interactions (12, 13). Due to the coating contamination of RIA and the false positives caused by non-specific and staggered reactions in ELISA, surface plasmon resonance (SPR), fluorescence activated cell sorting (FACS), bio-layer interferometry (BLI), cryogenic electron microscopy (cryo-EM) and other technologies are often used to detect the specificity of antibodies more accurately. However, some of these experimental methods are labor intensive, time-consuming, and costly. In addition, these experimental methods are unsuitable for large-scale high-throughput antibody screening.

On the other hand, the increasing availability of experimental data of antibody-antigen interaction provides valuable guidance for the development of computational approaches. The International ImmunoGeneTics (IMGT) information system is the most well-known immunity-related database that integrates sequence, genome, and structure immunogenetic data (14). Other sequence databases such as Database of ImmunoGlobulins with Integrated Tools (DIGIT) (15), abYsis (16), iReceptor (17), and Observed Antibody Space (OAS) (18) also source a large amount of sequencing data indicating antibody-antigen interaction. The Immune Epitope Database (IEDB) is established mainly for epitopes (19). The experimental data on antibody and T cell epitopes in the context of disease, allergy, autoimmunity and transplantation provides a reference for antibody design and immunotherapy development. Among these antibody-related databases, the structural antibody database (SAbDab) collects all the available antigen-antibody complexes in the Protein Data Bank (PDB) (20). Various types of antigens with their binding antibodies have provided insights for understanding the generic mechanisms of antigen-antibody binding. In contrast to SAbDab, the Coronavirus antibody database (CoV-AbDab) collected antibodies that bind to at least one beta coronavirus (21). Up to now (Version of July 2022), CoV-AbDab has included approximately 10,000 entries, which are valuable for the fundamental research of SARS-CoV-2 and the development of the corresponding vaccines and drugs.

Thanks to the increasingly available experimental data, several computational approaches for computational antibody design have been developed. Molecular docking is a classical method for predicting antibody-antigen binding mode and relative positions. However, molecular docking is often computationally expensive, especially when dealing with flexible molecules such as antibodies (22). The emergence of epitope or paratope prediction tools based on structural or sequence features, such as PECAN (23), BepiPred2.0 (24), and Epipred (25), greatly reduces the search space of docking. Many software or tools can directly predict antibody-antigen interactions. Lim et al. predicted the binding of PD-1 and CTLA-4 antibodies by training a convolutional neural network (CNN) with complementarity-determining region (CDR) sequence features (26). By using the multi-head attention network with position-embedding of CDRs, Wang et al. developed a model which can accurately distinguish the antibodies binding to the SARS-CoV-2 S protein and influenza HA (27). These computational methods provide alternative choices for early screening and effectively supplement experimental methods.

Still, the aforementioned computational methods have issues to be improved: (i) Most of them require the structural information of antigens or antibodies which are hard to obtain; (ii) These methods are often implemented for one specific antigen, and therefore are not widely applicable; (iii) The published algorithms or tools did not have a user-friendly interface for non-specialists to use. To this end, we proposed AbAgIntPre, an online tool to predict the interactions between antibodies and antigens based only on the sequence features. AbAgIntPre combines the composition of k-spaced amino acid pairs (CKSAAP) encoding and CNN deep learning framework for an efficient prediction of antibody-antigen interactions. AbAgIntPre enables general antigen-antibody interaction prediction by capturing various types of antigens. We also established a specific interaction prediction model for coronavirus, including severe acute respiratory syndrome coronavirus 1 (SARS-CoV-1) and SARS-CoV-2. For the convenience of the community, we have implemented AbAgIntPre as a web server with a friendly interface. Users can freely select the generic model and SARS-CoV-specific model to meet their needs.

## Methods

### Datasets

**Figure 1:**
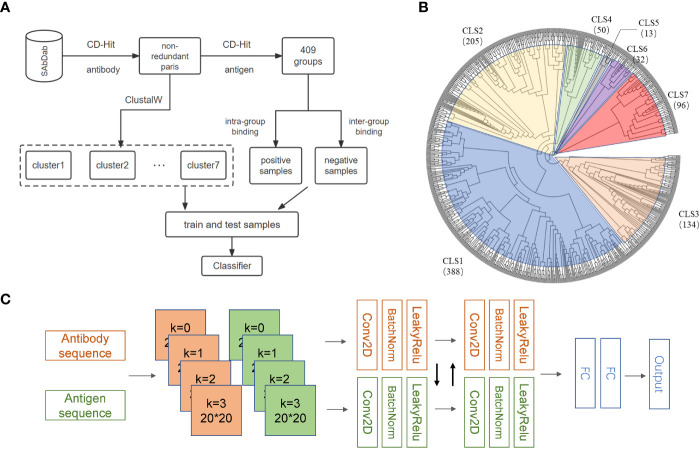
Overview of AbAgIntPre. **(A)** Flowchart of the generic prediction model of AbAgIntPre. **(B)** Clustering tree of 918 antigens in 408 subgroups. **(C)** The Siamese-like CNN framework of AbAgIntPre.

Many structures of antibodies and corresponding antigens are collected in the SAbDab database (20). We selected antibodies with heavy and light chain information and removed those complexes with antigenic sequences less than 50 amino acids (28). After applying this restriction, 1489 antibody-antigen complexes were retained. Considering the high specificity of the antibodies, we removed the sequence redundancy according to antibodies by applying CD-HIT (29) with a high sequence identity threshold of 0.98 and obtained 918 complexes. Because of the high specificity of the antibodies, we further divided the 918 complexes into 408 subgroups based on antigen sequences with a sequence identity threshold of 0.90. By the above procedures, we assumed that similar antibodies can bind similar antigens within the same subgroup, while the antigens and antibodies from different subgroups cannot bind to each other effectively (**Figure 1A**). As a result, we generated 3892 antibody-antigen pairs as the positive samples. Antigens and antibodies from different subgroups were randomly paired to form the negative samples, and the ratio of positive to negative samples was controlled to be 1: 1. Considering that the sequences of antibodies binding to different types of antigens are quite different, models may not perform satisfactorily in predicting the antigens which are quite different from those used in training. To this end, we used ClustalW (30) to establish the phylogenetic tree for antigens in the above 408 subgroups and divided these subgroups into seven clusters (**Figure 1B**). The training set and independent test set were generated from each cluster according to a ratio of 4:1 to ensure that no bias was introduced by the differences in antigen types.

We also collected antibodies binding to SARS-CoV and trained a SARS-CoV-specific predictor. SARS-CoV antibody data were collected from CoV-AbDab (21) and screened out the antibodies related to SARS-CoV-1 and SARS-CoV-2. Antibodies that could only bind to one SARS-CoV were taken as the positive samples, and those that could not bind to SARS-CoV were taken as the negative samples. A total of 9309 positive samples and 1710 negative samples were obtained, including 1965 positive samples of SARS-CoV-1, 7344 positive samples of SARS-CoV-2, 996 negative samples of SARS-CoV-2 and 714 negative samples of SARS-CoV-2.

### Sequence encoding

The previous literature has shown that the sequence compositions can effectively reflect the properties of antibodies such as specificity, stability, viscosity and immunogenicity. For example, Hebditch et al. predicted the solubility of antibodies by using a linear model with amino acid compositions and several other sequence features (31). Liaw et al. developed an algorithm for predicting amyloidogenesis of light chains of antibodies based on a random forest (RF) classifier with dipeptide composition (DPC) (32). Several analyses have demonstrated that there is also a preference of residues or motifs in epitopes of specific antibody (33–35). Kadam et al. developed a classifier for predicting antibody class(es) for epitopes with sequence composition features (36). El-Manzalawy et al. predicted flexible length linear B-cell epitopes by using support vector machine (SVM) with amino acid pairs encoding strategy (37).

In this study, we used CKSAAP as the preferred sequence composition-based encoding strategy. CKSAAP summarizes the frequency of k-spaced amino acid pairs normalized by all possible 400 kinds of pair combinations (38, 39), which reflected the amino acid composition of the antibodies and the residue preference of epitopes in antigens. To capture the characteristics of protein sequence more comprehensively, we calculated the results of each protein when *k* = 0,1,2,3. Therefore, each protein is represented as a 1600-dimensional vector.

We also compared the performance of CKSAAP encoding with those of the alternative encodings, including:

#### One-hot encoding

One-hot encoding is a typical sequence coding strategy in machine learning (ML). In one-hot encoding, we used a 20-bit vector of ‘0’ or ‘1’ to represent 20 kinds of amino acids, ‘1’ at each position denotes a specific amino acid, and ‘0’ for the rest of bits (40).

#### Position-specific scoring matrix encoding

In PSSM profiles, each residue in query protein is encoded as a 20-dimensional vector, in which each element reflects the conservation of 20 kinds of amino acids at corresponding position among a set of homologous sequences (41, 42). We generated the PSSM profiles of all the antigens and antibodies by applying PSI-BLAST (43) search against the NR90 database with three iterations. The e-value cutoff for including sequences in the profiles was set to 0.0001, and other parameters remained the default.

#### DPC encoding

DPC encoding uses a 400-dimensional vector to capture the character of the protein sequence (44). Each dimension corresponds to the frequency of a specific dipeptide combination in the query protein sequence.

#### Word2Vec embedding

In natural language processing, word2vec has been widely used to obtain the distributed representation of words (45, 46). The k-mers in protein sequences can be regarded as words in a document. Therefore, we trained a CBOW-based word2vec model with protein sequences in NR90 by using the *genism* of the python package. Each sequence was divided into several k-mers, and each k-mer was represented by a 64-dimensional embedding vector. In this study, we set k to 3 and set the window size to 4 to capture the context information.

### Deep learning framework

In this study, we used a Siamese-like CNN as the deep learning classifier to infer whether the query antigen and antibody can interact (**Figure 1C**). Specifically, our model mainly included three parts: input module, convolution module, and prediction module. In the input module, antigens or antibodies were coded by CKSAAP encoding scheme, and four channels correspond to four k-spaced values in CKSAAP. The convolution module further processed the encoded feature vectors. AbAgIntPre included two convolution modules, each of which consists of a batch normalization layer, convolution layer, rectified linear unit, and pooling. Two fully connected layers were used to map the learned distributed features to the sample label space and yield the probability of interaction between the given antigen and antibody.

We also compared the performance of our deep learning framework with those of several traditional ML models, including RF, SVM, Adaboost (ADA), logistic regression (LR), and multilayer perceptron (MLP). All the traditional ML models were implemented through the *sklearn* (https://scikit-learn.org/) of the python package.

### Performance evaluation

In this study, we used receiver operating characteristic (ROC) and precision-recall (PR) curves to measure the performance of the predictors. ROC and PR curves reflect the overall relationship between sensitivity and specificity and the overall relationship between precision and recall when different thresholds are applied. The definitions of sensitivity (i.e., recall), specificity, and precision are as follows:


$$Sensitivity=Recall=\frac{TP}{TP+FN}$$

$$Specificity=\frac{TN}{TN+FP}$$

$$Precision=\frac{TP}{TP+FP}$$

where TP, TN, FP, and FN denote the number of true positive, true negative, false positive, and false negative samples, respectively. The larger the area under the curve (AUC), the higher the prediction performance.

In addition, to test whether antigen-antibody binding could be simply determined by sequence similarity, we also compared our models with the well-established PSI-BLAST method on the independent test set. First, testing samples were aligned to training samples by PSI-BLAST (43). Then, we randomly combined each antigen and antibody with top 10 E-value in the PSI-BLAST results. If there was no combination appearing in the training positive set, then the query antigen-antibody pair was considered as non-interacting with zero prediction score. Otherwise, the prediction score could be calculated as follows:


$$PredictionScore_{PSI-BLAST}=\frac{100-\left(\left(Rank_{Ab}-1\right)\times \left(Rank_{Ag}-1\right)\right)}{100}$$

where *Rank_Ab_* and *Rank_Ag_* denote the ranking of E-value in PSI-BLAST results. The more similar the query antigen or antibody is to known sequences, the higher the prediction score.

## Results

### Establishment of AbAgIntPre generic prediction model

**Figure 2:**
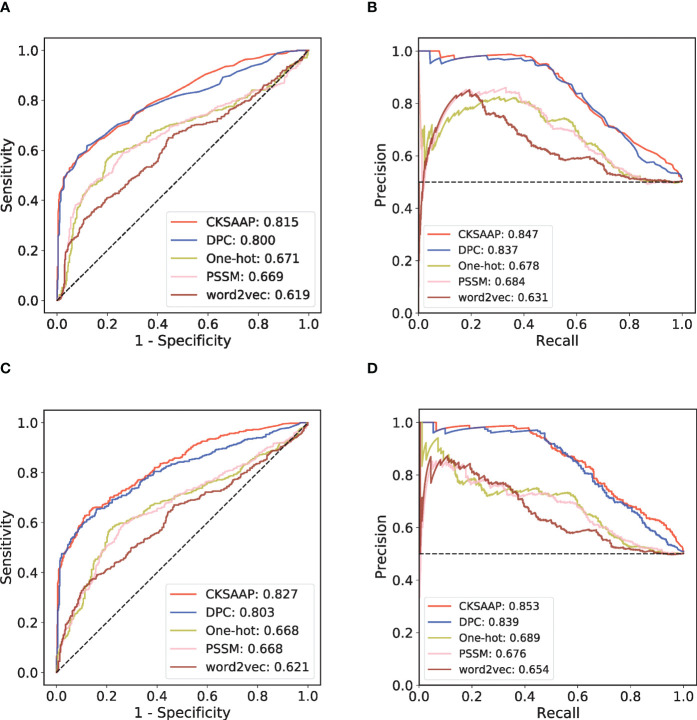
Performance comparison among different sequence encoding strategies in RF model and CNN model. **(A)** ROC curves of the five-fold cross-validation (CV) with RF model. **(B)** PR curves of the five-fold CV with RF model. **(C)** ROC curves of the five-fold CV with CNN model. **(D)** PR curves of the five-fold CV with CNN model.

By collecting various antigens and their corresponding antibodies in SAbDab, we have established a generic prediction model of antigen-antibody interaction. First, 918 high-quality antibody-antigen complexes were collected after data cleaning and redundancy removal. Then the 918 pairs of complexes were clustered into 408 subgroups. The high-quality positive and negative antibody-antigen pairs can be assigned based on this grouping, following the principle that similar antibodies bind similar antigens (**Figure 1B**; see Methods for details). In each cluster, we randomly divided the training set and the independent test set according to the ratio of 4: 1. We applied five-fold cross-validation (CV) on the training set. To select the sequence encoding method suitable for our study, we used the RF model to verify the one-hot, DPC, CKSAAP, PSSM, and word2vec encoding strategies in the five-fold CV (**Figures 2A, B**). **Figure 2A** shows that under the condition of low false positive rate, the true positive rates of word2vec, PSSM and one-hot encodings are significantly lower than those of DPC and CKSAAP encodings, which suggests that the encoding strategy based on amino acid composition is more suitable for dealing with antibody-antigen interaction issues.

**Figure 3:**
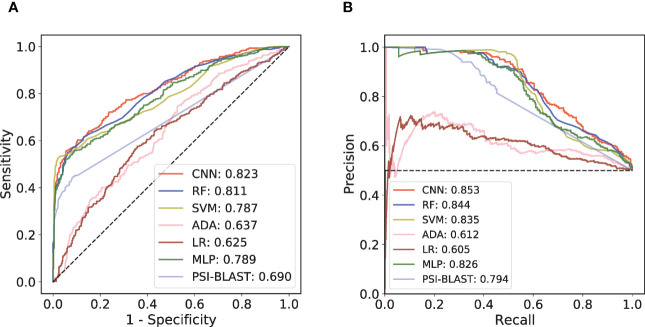
Performance comparison among different prediction models on independent test set. **(A)** ROC curves of the independent test. **(B)** PR curves of the independent test.

An independent test set that was not involved in the model’s training process was further introduced to validate the performance. We trained different ML models with the same training set and compared their performances on this independent test set. Considering that antibody-antigen interaction prediction belongs to the pair-input problem, among these ML models, we focused on the Siamese-like network which is commonly used in pair-input prediction problem (47–49). Our Siamese-like CNN ensured unbiased feature extraction by sharing the parameters between the two sub-networks. Although the results of CV based on RF model showed the superiority of CKSAAP encoding, we verified the applicability of CKSAAP encoding in CNN model through the same CV dataset used in RF model. As shown in **Figure 2**, the results with CNN (**Figures 2C, D**) agree well with those with RF models (**Figures 2A, B**). Since CKSAAP covers a broader range of compositional features than does the capture of only adjacent amino acids, we eventually adopted CKSAAP to encode the antigen and antibody sequences in the independent test and final training of the generic model. Independent test results show that the performance of our Siamese-like CNN model outperforms other traditional ML models (**Figure 3**). It is also worth noting that antigen-antibody interaction cannot be accurately judged simply by sequence similarity, as the true positive rate of PSI-BLAST was lower than those of some ML methods, especially than that of the CNN architecture when requiring lower false positive rate (**Figure 3**). Nonetheless, due to the scarceness of samples in rare antigen groups like the CLS5 covering only 13 antigens, our model was not sufficiently trained for such cases. We believe that the performance of our model could be further improved with the increasing experimental data.

### Establishment of AbAgIntPre SARS-CoV-specific prediction model

**Figure 4:**
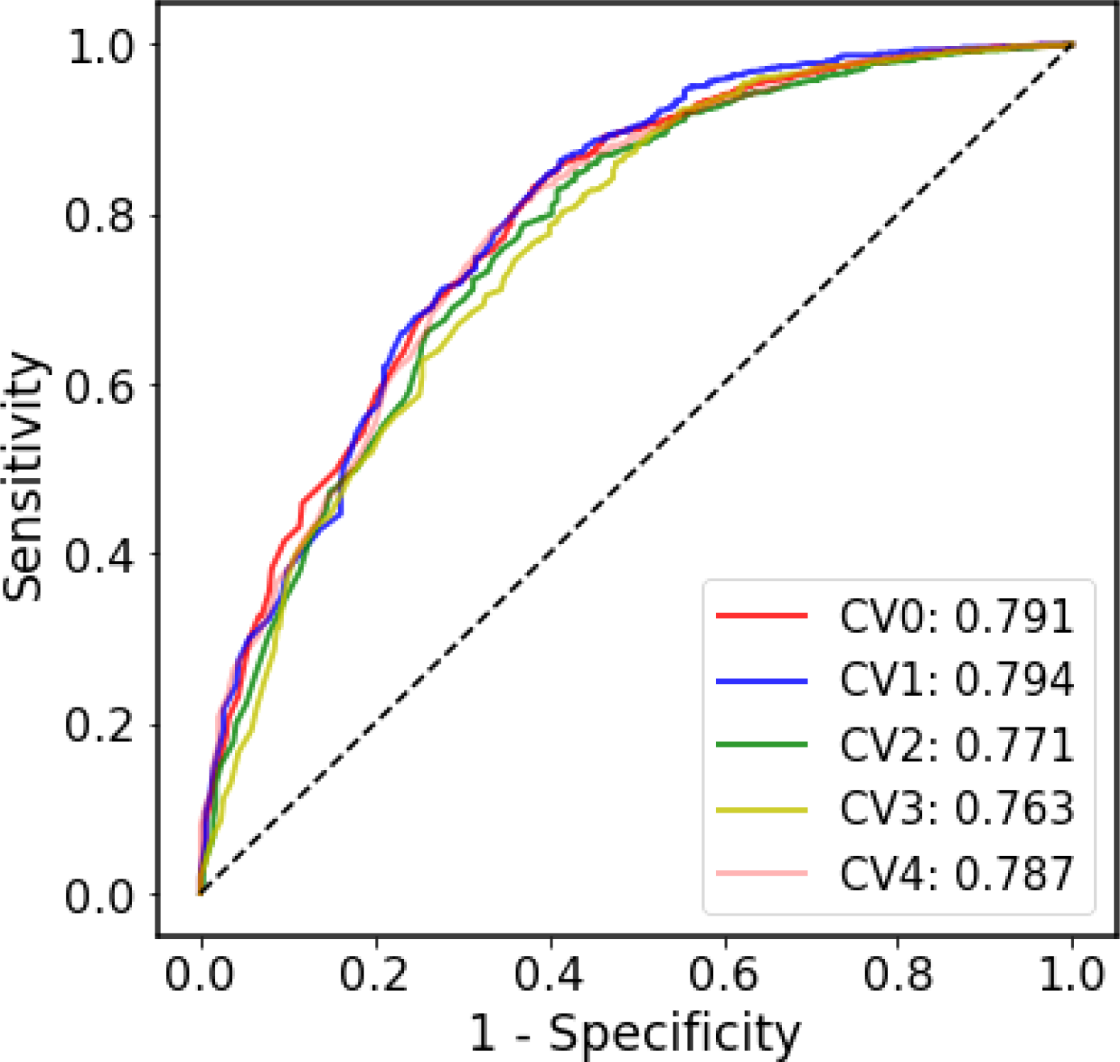
The ROC curves of the five-fold cross-validation on the SARS-CoV dataset.

Due to the highly contagious and sometimes lethal, the SARS-CoV pandemics present enormous challenges to medical care, economies, and social lives in 2003 and 2020-2022. Development of therapies for SARS-like coronavirus, especially pandemic SARS-CoV-2, is urgently needed. Effective characterizing and prediction for SARS-like coronavirus would assist vaccine and MAb therapy development and facilitate the elimination of the pathogens. In this study, we also established a SARS-CoV-specific prediction model to predict the probability that the antibodies bind SARS-CoV-1 and SARS-CoV-2. The SARS-CoV-specific prediction model was trained and tested based on the antibody-antigen pair annotations from CoV-AbDab. Due to the extreme disparity in the ratio of positive samples to negative samples, we used only ROC curve to evaluate the SARS-CoV-specific model since ROC curve is less affected by the unbalanced dataset. **Figure 4** shows that in the five-fold CV, the performance of each fold is equivalent to that of the generic model, and this result is acceptable considering the size and the unbalanced nature of the SARS-CoV dataset.

### Perturbation test on SARS-CoV-2 S protein antibodies

**Figure 5:**
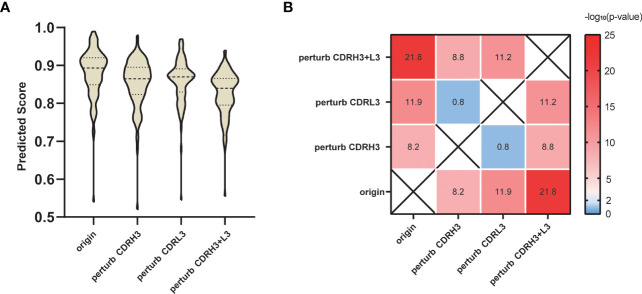
Perturbation test on SARS-CoV-2 S protein antibodies. **(A)** Prediction score distribution of SARS-CoV-2 S protein with antibodies under different perturbation strategies. **(B)** T-test results between different perturbation groups. Value in each heatmap cell represents the -log_10_ (p-value).

He et al. sorted SARS-CoV-specific single B cells to isolate 107 monoclonal antibodies from two recovered patients in COVID-19 (50). We collected these antibodies binding to SARS-CoV-2 S protein and then predicted the 107 interactions using our SARS-CoV-specific model. Considering that the CDRs, especially the CDRH3 and L3 loops play an important role in the antibody-specific recognition, we tested the validity of the prediction by randomly replacing the residues in CDRH3 and L3 loops. Specifically, for the above 107 antibodies, we applied three different perturbation tests: perturb CDRH3, perturb CDRL3 and perturb both CDRH3 and CDRL3. Taking perturb CDRH3 as an example, we randomly replaced every amino acid located in the CDRH3 loop with any of the other 19 amino acids to mimic the antibodies with perturbed functions. **Figure 5** shows that predicted scores of the perturbed antibodies significantly declined compared with the original antibodies. As expected, replacing CDRH3 or CDRL3 could severely reduce the prediction scores (p-value = 6.19E-09 and 1.13E-12, respectively), while the impact of the replacement of CDRH3 and CDRL3 together was the most significant (p-value = 1.67E-22).

### AbAgIntPre web server

To facilitate the community, we built a web server named AbAgIntPre with two models corresponding to the generic and the SARS-CoV-specific models, respectively. AbAgIntPre takes the FASTA sequences of the antigen, the antibody heavy chain and the antibody light chain as its input. The user can select the corresponding prediction model according to the antigen type. We also provide three levels of stringent thresholds, corresponding to the false positive rates of 1%, 5%, and 10%. The prediction result can be displayed directly on the web page, while is also supported to be sent *via* the provided E-mail address. We hope that the introduction of AbAgIntPre can reduce unnecessary experimental procedures and provide hypotheses and supplements for accelerating the development of antibody drugs.

## Discussion

The identification of antibody-antigen interaction is an essential problem in immunology and a prerequisite of antibody design and vaccine development. Although the experimental methods achieved the highest accuracy, these methods often need high cost of time, labor, and specific experimental conditions. Molecular docking based on protein structures is a common computational method for predicting antibody-antigen interactions (22). However, due to the difficulty in obtaining accurate structures of both antibody and antigen from sequences, predicting the interactions remains a difficult task. Mutual recognition of epitope and paratope is the key to specific binding of antigen and antibody. Many efforts have been devoted to predict the potential epitopes and paratopes based on ML methods (23–25), which provided insights into the characterization of antibodies and antigens through sequence features. Early studies have shown that the sequence components can well reflected the properties of antibodies and the amino acid preference of the epitopes (31, 32). In this study, we used CKSAAP encoding as the preferred and compared it with several popular sequence encoding strategies. The independent test results showed that CKSAAP encoding based on the sequence composition is superior to any other encoding strategies.

Although ML-based methods have been able to successfully predict potential paratopes and epitopes, direct prediction of antibody-antigen interaction remains a problem even if the epitopes or paratopes are known. The prediction of antibody-antigen interaction problem can be regard as a binary classification task. For well-known immunotherapy targets PD-1 and CTLA-4, the researchers have developed CNN model to predict interaction between antibodies and these two targets (26). Multi-head attention network was used to predict the binding of antibodies to SARS-CoV-2 S protein and influenza HA (27). Although these models performed well, they may not be able to predict unseen antigens due to their highly specific antigen scopes. For this reason, we established two prediction models, namely the generic prediction model and SARS-CoV-specific prediction model by using CNN architecture with CKSAAP sequence encoding strategy. Compared with the traditional ML methods, our results showed that the CNN architecture performs better both in the generic prediction model and SARS-CoV-specific prediction model. For the convenience of the community, we built the AbAgIntPre web server. Users can input the sequences of antigens and antibodies respectively to obtain the prediction results.

Although the performance of our prediction models is competitive, the applicable domain of our models is still limited. The generic prediction model achieves the purpose of prediction by learning the binding patterns of different types of antigens and antibodies, but it cannot accurately discern antibody-antigen pairs with subtle sequence variations. Therefore, the generic model is suitable for primary antibody-antigen pair screening, but not for precise antibody design. On the other hand, the SARS-CoV-specific model can more precisely predict whether an antibody-antigen pair can interact when the antibody or antigen sequences are similar to each other, but it is only applicable to SARS-CoV antibody-antigen pairs. Apparently, the quantity and diversity of antigens and antibodies in the training set directly affects the performance of our model in practical application. SAbDab contained all the antibody structures available in the PDB and we used these data to train the generic model (20). But the antibody/antigen coverage of SAbDab is still not fully satisfactory, and more exhaustive antibody-antigen interaction data with sequence annotations are required to improve the prediction. In addition to SAbDAb, we have also searched other popular immune-related databases (14, 16–19) for available training data. IMGT and Abysis are two well-known databases that provide a wealth of antibody data as well as a series of search tools. However, they mainly provide the germline sequences of antibodies and have no corresponding antigen information. IEDB houses abundant manually curated epitope data and most antibody-specific epitopes have been already included in the SAbDAb database. iReceptor and Observed Antibody Space collected NGS sequence data on B-cell receptors but there is no exact antigen information available. In summary, due to the limited data included in the public databases, there is still a prominent gap between the coverage of our training data and the real world. We believe that our models will perform better when more comprehensive and sizable antibody-antigen interaction data become available.

## Data availability statement

Publicly available datasets were analyzed in this study. This data can be found here: The original data are available at public database SAbDab (http://opig.stats.ox.ac.uk/webapps/newsabdab/sabdab/) and CoV-AbDab (http://opig.stats.ox.ac.uk/webapps/covabdab/). The processed datasets and source code of AbAgIntPre are available at: https://github.com/emersON106/AbAgIntPre. The web server of AbAgIntPre is available at http://www.zzdlab.com/AbAgIntPre.

## Author contributions

YZ and ZZ designed and supervised the study. YH performed the analysis, built the model and webserver, and drafted the manuscript. YZ and ZZ revised the manuscript. All authors contributed to the article and approved the submitted version.
